# Predicting mortality with machine learning & biomarkers: A cohort analysis from aerosolized β₂‐agonist for treatment of acute lung injury (ALTA) trial

**DOI:** 10.14814/phy2.70807

**Published:** 2026-03-22

**Authors:** Aaron M. Chase, Sultan Almuntashiri, Duo Zhang, Timothy W. Jones, Xianyan Chen, Payaningal R. Somanath, Kelli Henry, Andrea Sikora

**Affiliations:** ^1^ Department of Clinical and Experimental Therapeutics University of Georgia College of Pharmacy Augusta Georgia USA; ^2^ Department of Pharmacy University of Hail College of Pharmacy Ha'il Saudi Arabia; ^3^ Piedmont Hospital Atlanta Georgia USA; ^4^ Department of Statistics University of Georgia Franklin College of Arts and Sciences Athens Georgia USA; ^5^ Department of Biomedical Informatics University of Colorado Anschutz Medical Campus School of Medicine Aurora Colorado USA; ^6^ University of Georgia College of Pharmacy Augusta Georgia USA

**Keywords:** acute respiratory distress syndrome, artificial intelligence, biomarkers, critical care, machine learning, mortality

## Abstract

Laboratory‐guided methods have the potential to provide robust mortality prediction for acute respiratory distress syndrome (ARDS) which could improve timely intervention. The objective of this investigation was to predict mortality using regression and machine learning techniques using biomarkers linked to ARDS pathophysiology, including matrix metalloproteinase‐3 (MMP‐3) and club cell‐secretory protein‐16 (CC16), and general inflammatory biomarkers. 89 adult patients from the “Aerosolized β₂‐agonist for treatment of acute lung injury” (ALTA) trial were randomly separated into training (*n* = 53), validation (*n* = 8), and test (*n* = 28) sets. Logistic regression and supervised machine learning (ML) models were developed. In total, 20 ICU predictors including baseline characteristics (age, sex, APACHE III score, sepsis, vasoactive agent, PaO_2_/FiO_2_) and baseline and serial biomarkers were included. The primary outcome was area under the receiver operating characteristic (AUROC) for 90 day mortality. Random Forest, Support Vector Machine (SVM), and XGBoost achieved AUROCs of 0.917, 0.705, and 0.955, respectively. Stepwise regression achieved an AUROC of 0.508. For the highest performing model (XGBoost), MMP3‐based variables were the most important features. ML had high predictive ability for 90‐day mortality, and MMP‐3 demonstrates moderate‐to‐high feature importance in ML models. These findings support using pathophysiology‐derived biomarkers in ML models for ARDS prediction.

## 1 INTRODUCTION

The clinical heterogeneity of acute respiratory distress syndrome (ARDS) often results in missed or delayed diagnosis with up to two thirds of diagnoses being missed upon meeting clinical diagnostic criteria (Bellani et al., 2016). Delay in diagnosis prevents intervention in the early, exudative phase of ARDS when proven interventions are likely most effective (Bellani et al., 2016; Matthay et al., 2019; Sinha & Calfee, 2019; Thompson et al., 2017; Zhao et al., 2017). In addition to clinical presentation, the phenotypic heterogeneity of ARDS leads to differential treatment response to different interventions (Bellani et al., 2016). Quantitative, biomarker‐based approaches have been proposed as a means of improving diagnostic strategies and tailoring patient‐specific therapies (Matthay et al., 2019).

Previously, post‐hoc analyses of large, randomized controlled trials have shown that approaches using phenotypes described as hyper‐ and hypo‐inflammatory have shown variations in treatment response (Sinha & Calfee, 2019; Wilson & Calfee, 2020). Recently, latent‐class analysis revealed dexamethasone treatment effect occurs only in hyperinflammatory subtypes of COVID‐19 ARDS. (Sinha et al., 2021) Additionally, Calfee et al. found simvastatin was associated with improved survival in the hyperinflammatory phenotype, which was not observed in the hypo‐inflammatory phenotype or the combined groups (Calfee et al., 2018), while Famous et al. observed benefit from conservative fluid management in ARDS in the non‐inflammatory phenotype but worse outcomes with conservative fluid management in the inflammatory phenotype (Famous et al., 2017). Moreover, machine learning approaches to ARDS phenotyping have also shown novel treatment response patterns (Calfee et al., 2014; Grunwell et al., 2023; Singhal et al., 2021). Taken together, these investigations show machine learning as a potentially promising approach to identifying novel patterns within the clinical heterogeneity (Bhavani et al., 2022; Zhao et al., 2017); however, it is notable that most of the biomarkers used to date are relatively non‐specific to the ARDS process and may be elevated in critical illness, regardless of the presence of ARDS (Chase et al., 2022; Garcia‐Laorden et al., 2017; van der Zee et al., 2020; Wilson & Calfee, 2020).

Recently, several studies have pointed towards the potential of pathophysiology‐derived biomarkers for aiding in ARDS diagnosis, including club cell secretory protein 16 (CC16) and matrix metalloproteinase‐3 (MMP3) (Almuntashiri et al., 2022; Artham et al., 2019; Artham et al., 2020; Davey et al., 2011; Fligiel et al., 2006; Gelzo et al., 2022; Nelson et al., 2000; Nerusu et al., 2007; Pardo et al., 2016; Warner et al., 2001; Yamashita et al., 2016). In this analysis of samples from the study Randomized, placebo‐controlled clinical trial of an aerosolized β₂‐agonist for treatment of acute lung injury (ALTA) (National Heart, Lung, and Blood Institute Acute Respiratory Distress Syndrome (ARDS) Clinical Trials Network, 2011), we aimed to employ both traditional and machine learning techniques to develop and validate mortality prediction models that incorporate lung‐specific biomarkers. We hypothesized that machine learning techniques would have strong performance and would include biomarkers as significant predictor variables.

## 2 MATERIALS AND METHODS

### 2.1 Study design

This was an analysis of 89 adult patients with ARDS derived from the randomized, placebo‐controlled clinical trial of an aerosolized β₂‐agonist for the treatment of acute lung injury (ALTA) (National Heart, Lung, and Blood Institute Acute Respiratory Distress Syndrome (ARDS) Clinical Trials Network, 2011). This trial was selected due to the availability of biospecimens linked to relevant patient data in the context of a major, randomized, clinical trial for ARDS. The included samples were based on the availability from the National Heart, Lung, and Blood Institute's Biological Specimen and Data Repository Information Coordinating Center (BioLINCC) repository. The primary outcome was to determine the performance of each machine learning model for the prediction of 90‐day mortality compared to standard stepwise regression using area under the receiver operating characteristic (AUROC). Secondary outcomes included the change in predictive value after removal of MMP3 and CC16 variables from each model and identifying variables with the highest feature importance in each machine learning model. This project was deemed exempt by the University of Georgia Institutional Review Board (IRB) under PROJECT00006637 and procedures were followed in accordance with the Helsinki Declaration of 1975. Additionally, this manuscript is compliant with the Transparent Reporting of a multivariable prediction model for Individual Prognosis Or Diagnosis (TRIPOD) guidelines (Supplemental Digital Context–Appendix SI).

### 2.2 Biosamples

Plasma samples and coded data sheets from patients enrolled in ALTA were obtained from BioLINCC. Patient samples were stored frozen at −80°C. Plasma cytokine measurement of interleukin‐1 beta (IL‐1β), interleukin‐6 (IL‐6), monocyte chemoattractant protein‐1 (MCP‐1), tumor necrosis factor receptor superfamily member 1A (TNFRSF1A), C‐reactive protein (CRP), interleukin‐10 (IL‐10), interleukin‐8 (IL‐8), interferon‐gamma inducible protein (IP‐10), and lipopolysaccharide (LPS)‐binding protein (LBP) were measured in duplicate by this investigative team by human magnetic bead‐based multiplex assay (R&D Systems, Catalog #LXSAHM, Minneapolis, MN) according to the manufacturer's instructions. Plasma matrix metallopeptidase 3 (MMP‐3) concentration was measured in duplicate using an enzyme‐lined immunosorbent assay (ELISA) kit (R&D Systems, Catalog # DY513, Minneapolis, MN) according to the manufacturer's instructions.

### 2.3 Clinical trial number

Not applicable.

### 2.4 Statistical analysis

Prior to analysis, outliers were identified and removed using Tukey's Hinges with a *k*‐value of 3 or higher and a threshold value for each cytokine based on a literature search of maximum observed values (IL‐6 >5000 pg/mL, MCP‐1 >10,000 pg/mL, Il‐8 >6000 pg/mL, IP‐10 >10,000, and MMP3 s >1000 ng/mL). Missing data were imputed by the k‐nearest neighbors' method. After removal of outliers and imputation, the dataset was randomly separated into training (*n* = 53), validation (*n* = 8), and test (*n* = 28) sets. Due to the data being highly imbalanced in the training set (survival: 43, death: 10), Synthetic Minority Oversampling Technique (SMOTE) was applied to up‐sample and randomly down‐sample the training set. (Chawla et al., 2002) After resampling the training set was comprised of survival: 43 and death: 34. Due to the hypothesis‐generating nature of this study, a sample size estimation was not calculated, and all eligible patients from the available database were included to maximize the statistical power of the predictive models developed.

A total of 20 predictors were chosen based on a literature review and availability in the ALTA data set. The predictors included baseline clinical characteristics (age, sex, APACHE III score, sepsis, vasoactive agent, PaO_2_/FiO_2_), baseline biomarkers (day 0 MMP‐3, CC16, IL‐1β, IL‐6, MCP‐1, TNFRSF1A, CRP, IL‐10, IL‐8, IP‐10, and LBP), and serial biomarkers (Day 3 MMP‐3, Day 3 CC16, change in MMP‐3). Stepwise regression was conducted using backward selection, iteratively removing the least statistically significant predictor from a predictive model until an optimal subset of variables was identified. Then, three machine learning methods were applied to the entire variable list: Random Forest (RF), Support Vector Machine (SVM), and XGBoost. The complete variable list available from BioLINCC's is provided in Table S1.

Models were then fit using the training set and parameters were selected with grid search using the validation set. Results of the model performance are reported using the test set. For XGBoost, feature importance was measured as the frequency a feature was used in the trees. For Random Forest, feature importance was measured by the mean decrease in node impurity. For XGBoost, we utilized Gain to calculate the feature importance, which signifies the average improvement in accuracy achieved by splits associated with the feature. Because 10 different models for each were used on each imputed dataset, 10 different feature importance lists were generated. To determine the effect of variable absence on model performance, MMP‐3 and CC16 variables were removed from each model. One iteration without MMP‐3 variables (Day 0 MMP‐3, Day 3 MMP‐3, change in MMP‐3) and another without MMP‐3 and CC16 (Day 0 CC16, Day 3 CC16) variables. To compare performance of the models, area under the receiver operating characteristic (AUROC) was calculated and compared using DeLong test. Positive predictive value (PPV), negative predictive value (NPV), specificity, and sensitivity were calculated for each model.

All analyses were completed in Python, and significance was assessed at an alpha of 0.05.

## 3 RESULTS

A total of 89 patients were included with a mortality rate of 22 patients (25%) by 90 days. Of the 22 deaths, 15 (68%) occurred within 30 days. Non‐survivors at 90 days had a significantly higher APACHE III score (105 vs. 84, *p* = 0.002) and were significantly older (62.0 vs. 46.8 years, *p* < 0.001). Non‐survivors were also numerically more likely to have sepsis (41% vs. 21%, *p* = 0.1) and had less vasopressor use at trial enrollment (36% vs. 61%, *p* = 0.07). Non‐survivors had significantly higher baseline TNFRSF1A (8437 ng/mL vs. 4790 ng/mL, *p* = 0.002), IL‐1B (16.5 pg/mL vs. 12.9 pg/mL, *p* < 0.001), IL‐10 (55.6 pg/mL vs. 29.6 pg/mL, *p* = 0.005), and IL‐8 (129.3 pg/mL vs. 33.4 pg/mL, *p* < 0.001). Day 0 MMP‐3, Day 0 CC16, and Day 3 CC16 were similar between survivors and non‐survivors. Day 3 MMP‐3 was significantly higher in non‐survivors (42.5 ng/mL vs. 20.1 ng/mL, *p* = 0.002), and non‐survivors had a markedly higher increase in MMP‐3 from day 0 to 3 (27.7 vs. 6.8, *p* < 0.001). Table 1 summarizes baseline characteristics.

**TABLE 1:** Demographic characteristics and patient outcomes of patients used in model building.

Characteristic	Total (*n* = 89)	Mortality at 90 days (*n* = 22)	Survival at 90 days (*n* = 67)	*p*‐value
Co‐variates				
Sex, male	47 (52.8)	13 (59.1)	34 (50.8)	0.700
Age, years	50.55 *±* 15.74	62.00 *±* 10.68	46.79 *±* 15.36	<0.001
APACHE III	89.23 *±* 27.63	105.24 *±* 27.06	84.14 *±* 25.99	0.002
PaO_2_/FiO_2_	157.18 ± 63.09	160.38 ± 53.51	156.17 ± 66.20	0.8
Vasopressors	49 (55.06)	8 (36.36)	41 (61.19)	0.07
Sepsis	23 (25.84)	9 (40.91)	14 (20.90)	0.1
MMP3 day 0, ng/mL	13.65 ± 10.02	14.75 ± 8.61	13.30 ± 10.71	0.6
MMP3 day 3, ng/mL	25.62 ± 30.09	42.45 ± 45.49	20.09 ± 20.56	0.002
MMP3 day 0 to 3 change, ng/mL	11.96 ± 26.46	27.71 ± 41.93	6.80 ± 16.26	0.001
CC16 day 0, ng/mL	62.53 ± 57.84	74.54 ± 60.28	58.58 ± 56.93	0.3
CC16 day 3, ng/mL	69.07 ± 89.93	87.07 ± 82.02	63.17 ± 92.19	0.3
IL‐1B day 0, pg/mL	13.80 ± 4.33	16.49 ± 5.73	12.93 ± 3.39	<0.001
IL‐6 day 0, pg/mL	309.51 ± 572.33	357.16 ± 495.22	293.86 ± 598.09	0.7
MCP‐1 day 0, pg/mL	542.05 ± 712.92	630.21 ± 715.83	513.11 ± 714.97	0.5
TNFRSF1A day 0, ng/mL	5592.31 ± 4819.58	8437.56 ± 5928.40	4790.89 ± 4056.02	0.002
CRP day 0, pg/mL	74301.54 ± 21700.99	69876.62 ± 26509.78	75754.50 ± 19890.52	0.4
IL‐10 day 0, pg/mL	35.88 ± 37.70	55.61 ± 64.27	29.60 ± 21.07	0.005
IL‐8 day 0, pg/mL	57.13 ± 117.39	129.37 ± 215.31	33.41 ± 36.21	<0.001
IP‐10 day 0, pg/mL	338.16 ± 800.39	441.13 ± 866.89	304.35 ± 781.25	0.5
LBP day 0, pg/mL	1.36e+06 ± 3.22e + 05	1.32e+06 ± 4.10e + 05	1.38e+06 ± 2.89e + 05	0.4
Outcomes				
90‐day mortality	22 (24.72)	22 (100)	0	‐
30‐day mortality	15 (16.85)	15 (68.18)	0	‐
ICU free days through 28 days	13.92 ± 9.21	3.95 ± 7.05	17.19 ± 7.30	<0.001
Ventilator free days through 28 days	14.85 ± 10.30	2.22 ± 6.15	19.00 ± 7.67	<0.001
Organ failure free days through 28 days	15.66 ± 10.26	6.64 ± 7.25	18.63 ± 9.35	<0.001

*Note*: Data are presented as *n* (%) or mean ± standard deviation (SD) unless otherwise stated.

Abbreviations: APACHE III, Acute Physiology and Chronic Health Evaluation III; CRP, C‐reactive protein; ICU, intensive care unit; IP‐10, human interferon‐inducible protein 10; LBP, lipopolysaccharide‐binding protein; MCP‐1, monocyte chemoattractant protein‐1; MMP‐3, matrix metalloproteinase‐3; TNFRSF1A, tumor necrosis factor receptor superfamily member 1A.

A regression model including all variables was developed (Table S1) in addition to stepwise and machine learning‐based models. The stepwise regression model demonstrated the lowest AUROC and poor predictive performance (Table S2). Among the machine learning models, XGBoost had the highest AUROC (0.955), followed by Random Forest (0.917) and SVM (0.705). AUROC, predictive values, sensitivity, and specificity are summarized in Table 2. AUROC curves for all models are depicted in Figure S1.

**TABLE 2:** Model performance comparison.

	AUROC	Sensitivity (recall)	Specificity	PPV (precision)	NPV
Stepwise	0.508	0.333	0.773	0.286	0.810
Random Forest	0.917	0.5	0.955	0.750	0.875
Support vector machine	0.705	0.167	0.864	0.250	0.792
XGBoost	0.955	0.5	0.955	0.750	0.875
Exclusion of lung specific biomarkers					
Random Forest without MMP‐3	0.765	0.167	0.955	0.50	0.808
Random Forest without MMP‐3 and CC16	0.826	0.667	0.818	0.5	0.900
Support vector machine without MMP‐3	0.689	0.167	0.864	0.250	0.792
Support vector machine without MMP‐3 and CC16	0.705	0.167	0.818	0.200	0.818
XGBoost without MMP‐3	0.792	0.00	0.955	0.00	0.778
XGBoost without MMP‐3 and CC16	0.701	0.00	0.955	0.00	0.778

Abbreviations: AUROC, area under the receiver operating characteristic; NPV, negative predictive value; PPV, positive predictive value.

**FIGURE 1:**
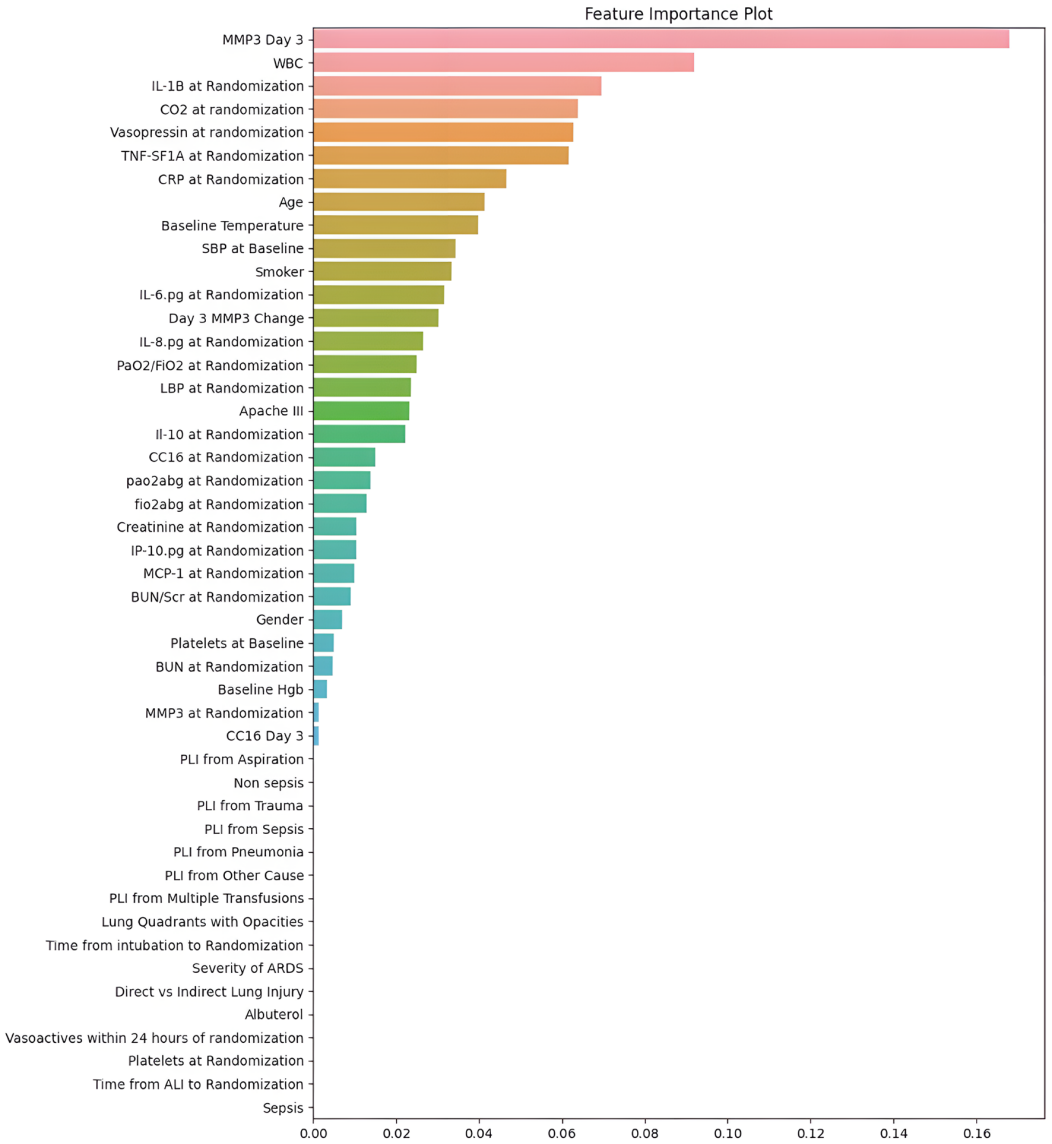
Feature Importance for Random Forest.

**FIGURE 2:**
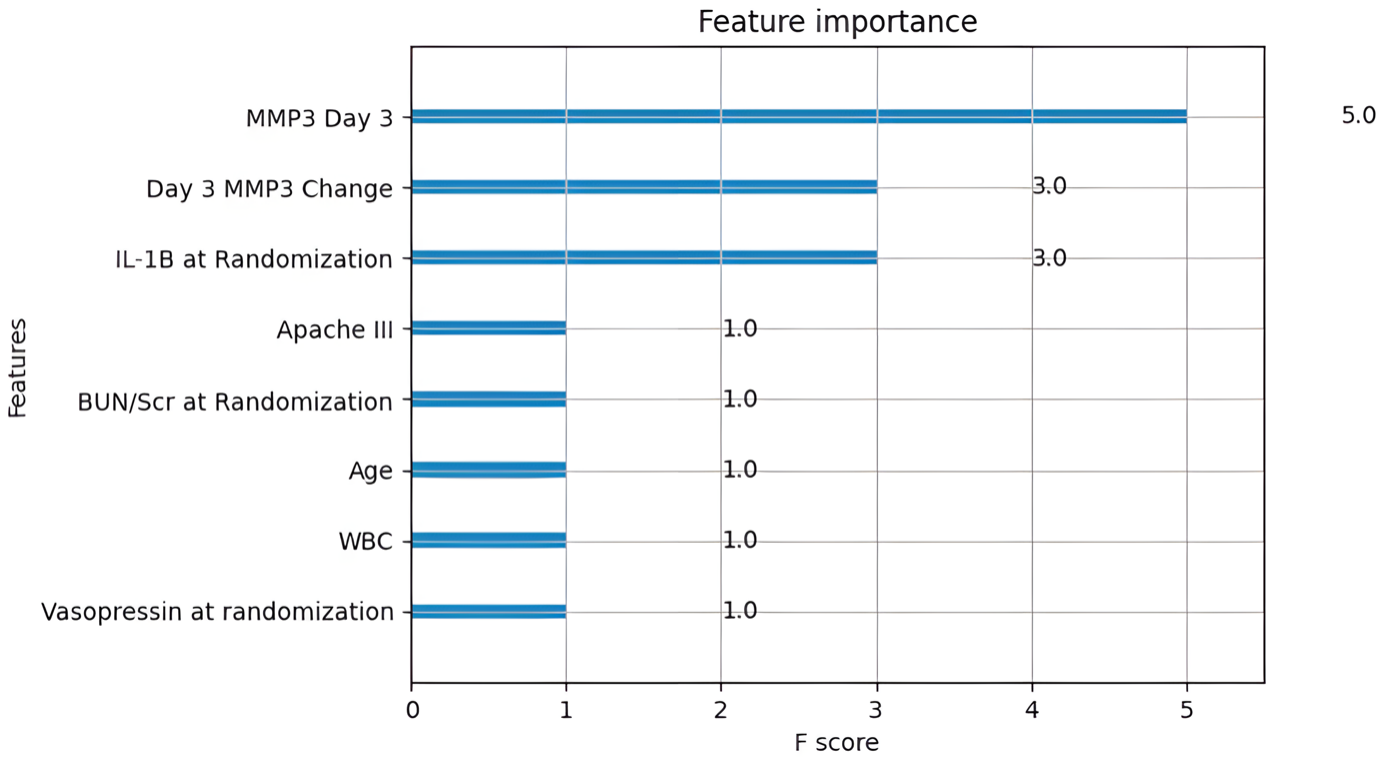
Feature Importance for XGBoost.

The variable with the highest feature importance in the XGBoost model (Figure 1) was Day 3 MMP‐3 followed by change in MMP‐3, baseline IL‐1β, APACHE III score, BUN/SCr ratio, age, white blood cell count, and vasopressor use. Day 3 MMP‐3 was also the variable with the highest feature importance in the Random Forest model (Figure 2). Conversely, in the lowest performing SVM model, the highest ranking variables were IL‐10 and clinical variables such as age, smoking status, and sex with several biomarkers, including MMP‐3, producing negative feature importance (Figure S2).

When both MMP3 and CC16 variables were removed from the XGBoost model, there was a significant reduction in the AUROC for prediction of 90‐day mortality (0.955 vs. 0.701, *p* = 0.01). Removal of these variables from the Random Forest model resulted in a non‐significant decrease in AUROC (0.917 vs. 0.826, *p* = 0.18). No change was observed with removal of both biomarkers from the SVM model (0.705 vs. 0.705, *p* = 1.00). There was no significant change when CC16 or MMP3 variables alone were removed from any model individually. A full comparison is reported in Table S3, and AUROCs are depicted in Figures S3 and S4.

## 4 DISCUSSION

Our analysis represents one of the first machine learning prediction models employing ARDS pathophysiology derived biomarkers, including MMP‐3 and CC16, from a randomized clinical trial. Machine learning models had a high predictive power to identify patients at risk of 90‐day mortality using predominantly biomarker‐based data. Both a full and stepwise regression showed similar AUROC and high PPV and NPV. XGBoost displayed more robust predictive capabilities compared to Random Foreset or Support Vector Machine models. These analyses revealed significant differences with regard to predictor variables that favor further exploration of artificial intelligence for biomarker‐based mortality prediction in ARDS.

Previous studies using large ARDS databases have primarily focused on mortality prediction and phenotype identification through unsupervised machine learning techniques such as latent class analysis and clustering algorithms. These approaches have consistently revealed two biologically distinct ARDS phenotypes that differ in response to interventions like lung‐protective ventilation, corticosteroids (e.g., dexamethasone), and fluid management.^9–11^ In contrast, our study employs supervised machine learning methods, shifting the focus from phenotype discovery to outcome prediction. This approach allows for the integration of ARDS pathophysiology derived biomarkers—namely MMP‐3 and CC16—which are more directly tied to the specific disease process. While earlier studies examined these biomarkers using traditional statistical analyses, we expand on that work by applying multiple supervised machine learning models, which highlights the most influential variables through feature importance analysis. This strategy enhances the identification of potentially actionable biomarkers and key drivers of disease progression. Ultimately, combining both supervised and unsupervised approaches may offer a more comprehensive understanding of ARDS heterogeneity and support the development of personalized therapeutic strategies.

MMP‐3 and CC16 have both been explored as prognostic biomarkers in ARDS, but their utility appears to differ based on our findings. MMP‐3, which plays a role in the early, exudative phase of ARDS, has shown promise as a prognostic marker and potential therapeutic target. Prior analyses, including data from the ALTA trial, demonstrated strong predictive performance with AUROCs of 0.77 and 0.74 for day 3 levels and changes in MMP‐3, respectively. MMP‐3 has also shown prognostic value in related conditions such as COVID‐19 ARDS and pneumonia. Our study complements these findings, revealing that MMP‐3 consistently ranked high in feature importance across both regression and multiple machine learning models. Further, removal of MMP‐3 variables from models dropped the predictive ability of the respective model. This underscores its relevance in predictive modeling and its potential as a focus for future research.

In contrast, CC16 did not demonstrate strong predictive value in our analyses. Despite prior studies suggesting prognostic utility with AUROCs ranging from 0.67 to 0.78 using simpler models, CC16 showed low feature importance in both standard regression and advanced machine learning approaches. This may be due to the inclusion of more influential clinical variables, as well as the anatomical origin of CC16 in the terminal bronchioles, which may be less relevant to the alveolar‐centered pathology of ARDS. Our findings suggest that while CC16 may have some lung specificity, biomarkers like MMP‐3, which is more directly linked to ARDS pathophysiology, may offer greater value in mortality prediction models.

Our study has several limitations. While the sample size is average by critical care standards, it remains small for machine learning applications. The dynamic nature of ICU patients is also not fully captured, as all predictions were based solely on data from the first 24–72 h of ICU admission. Incorporating longitudinal changes and exploring unsupervised learning techniques may offer additional insights. Finally, we observed that MMP‐3 had a negative feature importance in the SVM model, in contrast to its strong performance in tree‐based models like Random Forest and XGBoost. This discrepancy likely stems from differences in how feature importance is calculated: SVM relied on permutation importance, which can undervalue highly correlated features and is less stable in small datasets. In contrast, tree‐based models benefit from ensemble methods and bootstrapping, which help stabilize importance rankings and may be better suited for datasets of this size. Moreover, while leveraging data from a randomized controlled study, the present work lacks external validation using a separate dataset, and it does not address how such a prediction metric could be operationalized in clinical practice, both of which warrant further investigation. Additionally, this study design allowed us to examine the impact of MMP‐3 and CC16 using supervised machine learning methods; however, it did not attempt to identify new biomarkers, which may be a future application of machine learning methodology.

## 5 CONCLUSION

In this study, supervised machine learning methods had high predictive value for 90‐day mortality using a combination of lung‐specific biomarkers and clinical variables. Feature importance analysis consistently ranked MMP‐3–related variables among the most influential, reinforcing the potential of this biomarker in prognosticating ARDS outcomes and disease progression. Future application of unsupervised machine learning to larger datasets, integrated with biomarkers central to ARDS pathophysiology, may facilitate the identification of clinically relevant phenotypes and actionable targets to improve patient outcomes.

## AUTHOR CONTRIBUTIONS

Aaron M. Chase, Timothy W. Jones, and Andrea Sikora conducted manuscript drafting, results interpretation, and critical revisions. Aaron M. Chase, Andrea Sikora, Duo Zhang, Payaningal R. Somanath, Xianyan Chen, Kelli Henry, and Timothy W. Jones provided manuscript review and revisions. Timothy W. Jones, Aaron M. Chase, and Andrea Sikora conducted laboratory analysis. Xianyan Chen conducted biostatistical analysis. Andrea Sikora, KH, Duo Zhang, and Payaningal R. Somanath provided project oversight, manuscript drafting, revisions, and results interpretation. Supplemental digital content is available for this article. Direct URL citations appear in the printed text and are provided in the HTML and PDF versions of this article on the journal's website (http://journals.lww.com/ccmjournal).

## FUNDING INFORMATION

This work was supported by the National Heart, Lung, and Blood Institute (NHLBI) grant R00HL141685 and R56HL163607 to DZ.

## CONFLICT OF INTEREST STATEMENT

The authors declare no conflicts of interest.

## CODE AVAILABILITY STATEMENT

The code used and/or analyzed during the current study is available from the corresponding author on reasonable request.

## ETHICS STATEMENT

This project was deemed exempt by the University of Georgia Institutional Review Board (IRB) under PROJECT00006637.

## Supporting information


Data S1.

## Data Availability

The datasets used and/or analyzed during the current study are available from the corresponding author on reasonable request.

## References

[phy270807-bib-0001] Almuntashiri, S. , Zhang, D. , Somanath, P. R. , & Sikora, A. (2022). MMP3 in severe COVID‐19: A biomarker or therapeutic target? Infectious Disorders Drug Targets, 23(1), e190622206159. 10.2174/1871526522666220619121539 PMC11042506 35726419

[phy270807-bib-0002] Artham, S. , Gao, F. , Verma, A. , Alwhaibi, A. , Sabbineni, H. , Hafez, S. , Ergul, A. , & Somanath, P. R. (2019). Endothelial stromelysin1 regulation by the forkhead box‐O transcription factors is crucial in the exudative phase of acute lung injury. Pharmacological Research, 141, 249–263. 30611853 10.1016/j.phrs.2019.01.006 PMC6532785

[phy270807-bib-0003] Artham, S. , Verma, A. , Newsome, A. S. , & Somanath, P. R. (2020). Patients with acute respiratory distress syndrome exhibit increased stromelysin1 activity in the blood samples. Cytokine, 131, 155086. 32272349 10.1016/j.cyto.2020.155086 PMC7416496

[phy270807-bib-0004] Bellani, G. , Laffey, J. G. , Pham, T. , Fan, E. , Brochard, L. , Esteban, A. , Gattinoni, L. , van Haren, F. , Larsson, A. , McAuley, D. F. , Ranieri, M. , Rubenfeld, G. , Thompson, B. T. , Wrigge, H. , Slutsky, A. S. , & Pesenti, A. (2016). Epidemiology, patterns of care, and mortality for patients with acute respiratory distress syndrome in intensive care units in 50 countries. JAMA, 315(8), 788–800. 26903337 10.1001/jama.2016.0291

[phy270807-bib-0005] Bhavani, S. V. , Semler, M. , Qian, E. T. , Verhoef, P. A. , Robichaux, C. , Churpek, M. M. , & Coopersmith, C. M. (2022). Development and validation of novel sepsis subphenotypes using trajectories of vital signs. Intensive Care Medicine, 48(11), 1582–1592. 36152041 10.1007/s00134-022-06890-z PMC9510534

[phy270807-bib-0006] Calfee, C. S. , Delucchi, K. , Parsons, P. E. , Thompson, B. T. , Ware, L. B. , & Matthay, M. A. (2014). Subphenotypes in acute respiratory distress syndrome: Latent class analysis of data from two randomised controlled trials. Lancet Respiratory Medicine, 2(8), 611–620. 24853585 10.1016/S2213-2600(14)70097-9 PMC4154544

[phy270807-bib-0007] Calfee, C. S. , Delucchi, K. L. , Sinha, P. , Matthay, M. A. , Hackett, J. , Shankar‐Hari, M. , McDowell, C. , Laffey, J. G. , O'Kane, C. M. , & McAuley, D. F. (2018). Acute respiratory distress syndrome subphenotypes and differential response to simvastatin: Secondary analysis of a randomised controlled trial. Lancet Respiratory Medicine, 6(9), 691–698. 30078618 10.1016/S2213-2600(18)30177-2 PMC6201750

[phy270807-bib-0008] Chase, A. , Almuntashiri, S. , Sikora, A. , & Zhang, D. (2022). Club cell secretory protein‐derived acute respiratory distress syndrome phenotypes predict 90‐day mortality: A reanalysis of the fluids and catheter treatment trial. Critical Care Explorations, 4(6), e0711. 35651737 10.1097/CCE.0000000000000711 PMC9150885

[phy270807-bib-0009] Chawla, N. V. , Bowyer, K. W. , Hall, L. O. , & Kegelmeyer, W. P. (2002). SMOTE: Synthetic minority over‐sampling technique. Journal of Artificial Intelligence Research, 16(1), 321–357.

[phy270807-bib-0010] Davey, A. , McAuley, D. F. , & O'Kane, C. M. (2011). Matrix metalloproteinases in acute lung injury: Mediators of injury and drivers of repair. European Respiratory Journal, 38(4), 959–970. 21565917 10.1183/09031936.00032111

[phy270807-bib-0011] Famous, K. R. , Delucchi, K. , Ware, L. B. , Kangelaris, K. N. , Liu, K. D. , Thompson, B. T. , & Calfee, C. S. (2017). Acute respiratory distress syndrome subphenotypes respond differently to randomized fluid management strategy. American Journal of Respiratory and Critical Care Medicine, 195(3), 331–338. 27513822 10.1164/rccm.201603-0645OC PMC5328179

[phy270807-bib-0012] Fligiel, S. E. , Standiford, T. , Fligiel, H. M. , Tashkin, D. , Strieter, R. M. , Warner, R. L. , Johnson, K. J. , & Varani, J. (2006). Matrix metalloproteinases and matrix metalloproteinase inhibitors in acute lung injury. Human Pathology, 37(4), 422–430. 16564916 10.1016/j.humpath.2005.11.023

[phy270807-bib-0013] Garcia‐Laorden, M. I. , Lorente, J. A. , Flores, C. , Slutsky, A. S. , & Villar, J. (2017). Biomarkers for the acute respiratory distress syndrome: How to make the diagnosis more precise. Annals of Translational Medicine, 5(14), 283. 28828358 10.21037/atm.2017.06.49 PMC5537109

[phy270807-bib-0014] Gelzo, M. , Cacciapuoti, S. , Pinchera, B. , De Rosa, A. , Cernera, G. , Scialò, F. , Comegna, M. , Mormile, M. , Fabbrocini, G. , Parrella, R. , Corso, G. , Gentile, I. , & Castaldo, G. (2022). Matrix metalloproteinases (MMP) 3 and 9 as biomarkers of severity in COVID‐19 patients. Scientific Reports, 12(1), 1212. 35075175 10.1038/s41598-021-04677-8 PMC8786927

[phy270807-bib-0015] Grunwell, J. R. , Rad, M. G. , Ripple, M. J. , Yehya, N. , Wong, H. R. , & Kamaleswaran, R. (2023). Identification of a pediatric acute hypoxemic respiratory failure signature in peripheral blood leukocytes at 24 hours post‐ICU admission with machine learning. Frontiers in Pediatrics, 11, 1159473. 37009294 10.3389/fped.2023.1159473 PMC10063855

[phy270807-bib-0016] Matthay, M. A. , Zemans, R. L. , Zimmerman, G. A. , Arabi, Y. M. , Beitler, J. R. , Mercat, A. , Herridge, M. , Randolph, A. G. , & Calfee, C. S. (2019). Acute respiratory distress syndrome. Nature Reviews. Disease Primers, 5(1), 18. 10.1038/s41572-019-0069-0 PMC6709677 30872586

[phy270807-bib-0017] National Heart, Lung, and Blood Institute Acute Respiratory Distress Syndrome (ARDS) Clinical Trials Network , Matthay, M. A. , Brower, R. G. , Carson, S. , Douglas, I. S. , Eisner, M. , Hite, D. , Holets, S. , & Kallet, R. H. (2011). Randomized, placebo‐controlled clinical trial of an aerosolized beta(2)‐agonist for treatment of acute lung injury. American Journal of Respiratory and Critical Care Medicine, 184(5), 561–568. 21562125 10.1164/rccm.201012-2090OC PMC3175548

[phy270807-bib-0018] Nelson, A. R. , Fingleton, B. , Rothenberg, M. L. , & Matrisian, L. M. (2000). Matrix metalloproteinases: Biologic activity and clinical implications. Journal of Clinical Oncology, 18(5), 1135–1149. 10694567 10.1200/JCO.2000.18.5.1135

[phy270807-bib-0019] Nerusu, K. C. , Warner, R. L. , Bhagavathula, N. , McClintock, S. D. , Johnson, K. J. , & Varani, J. (2007). Matrix metalloproteinase‐3 (stromelysin‐1) in acute inflammatory tissue injury. Experimental and Molecular Pathology, 83(2), 169–176. 17540368 10.1016/j.yexmp.2007.04.003 PMC2062514

[phy270807-bib-0020] Pardo, A. , Cabrera, S. , Maldonado, M. , & Selman, M. (2016). Role of matrix metalloproteinases in the pathogenesis of idiopathic pulmonary fibrosis. Respiratory Research, 17, 23. 26944412 10.1186/s12931-016-0343-6 PMC4779202

[phy270807-bib-0021] Singhal, L. , Garg, Y. , Yang, P. , Tabaie, A. , Wong, A. I. , Mohammed, A. , Chinthala, L. , Kadaria, D. , Sodhi, A. , Holder, A. L. , Esper, A. , Blum, J. M. , Davis, R. L. , Clifford, G. D. , Martin, G. S. , & Kamaleswaran, R. (2021). eARDS: A multi‐center validation of an interpretable machine learning algorithm of early onset acute respiratory distress syndrome (ARDS) among critically ill adults with COVID‐19. PLoS One, 16(9), e0257056. 34559819 10.1371/journal.pone.0257056 PMC8462682

[phy270807-bib-0022] Sinha, P. , & Calfee, C. S. (2019). Phenotypes in acute respiratory distress syndrome: Moving towards precision medicine. Current Opinion in Critical Care, 25(1), 12–20. 30531367 10.1097/MCC.0000000000000571 PMC6814152

[phy270807-bib-0023] Sinha, P. , Furfaro, D. , Cummings, M. J. , Abrams, D. , Delucchi, K. , Maddali, M. V. , He, J. , Thompson, A. , Murn, M. , Fountain, J. , Rosen, A. , Robbins‐Juarez, S. Y. , Adan, M. A. , Satish, T. , Madhavan, M. , Gupta, A. , Lyashchenko, A. K. , Agerstrand, C. , Yip, N. H. , … O'Donnell, M. R. (2021). Latent class analysis reveals COVID‐19–related acute respiratory distress syndrome subgroups with differential responses to corticosteroids. American Journal of Respiratory and Critical Care Medicine, 204(11), 1274–1285. 34543591 10.1164/rccm.202105-1302OC PMC8786071

[phy270807-bib-0024] Thompson, B. T. , Chambers, R. C. , & Liu, K. D. (2017). Acute respiratory distress syndrome. The New England Journal of Medicine, 377(19), 1904–1905. 10.1056/NEJMc1711824 29117492

[phy270807-bib-0025] van der Zee, P. , Rietdijk, W. , Somhorst, P. , Endeman, H. , & Gommers, D. (2020). A systematic review of biomarkers multivariately associated with acute respiratory distress syndrome development and mortality. Critical Care, 24(1), 243. 32448370 10.1186/s13054-020-02913-7 PMC7245629

[phy270807-bib-0026] Warner, R. L. , Beltran, L. , Younkin, E. M. , Lewis, C. S. , Weiss, S. J. , Varani, J. , & Johnson, K. J. (2001). Role of stromelysin 1 and gelatinase B in experimental acute lung injury. American Journal of Respiratory Cell and Molecular Biology, 24(5), 537–544. 11350822 10.1165/ajrcmb.24.5.4160

[phy270807-bib-0027] Wilson, J. G. , & Calfee, C. S. (2020). ARDS subphenotypes: Understanding a heterogeneous syndrome. Critical Care, 24(1), 102. 32204722 10.1186/s13054-020-2778-x PMC7092435

[phy270807-bib-0028] Yamashita, C. M. , Cybulskie, C. , Milos, S. , Zuo, Y. Y. , McCaig, L. A. , & Veldhuizen, R. A. (2016). The effect of matrix metalloproteinase‐3 deficiency on pulmonary surfactant in a mouse model of acute lung injury. Canadian Journal of Physiology and Pharmacology, 94(6), 682–685. 27096327 10.1139/cjpp-2015-0377 PMC5522962

[phy270807-bib-0029] Zhao, Z. , Wickersham, N. , Kangelaris, K. N. , May, A. K. , Bernard, G. R. , Matthay, M. A. , Calfee, C. S. , Koyama, T. , & Ware, L. B. (2017). External validation of a biomarker and clinical prediction model for hospital mortality in acute respiratory distress syndrome. Intensive Care Medicine, 43(8), 1123–1131. 28593401 10.1007/s00134-017-4854-5 PMC5978765

